# Multi-functionalized carbon dots as theranostic nanoagent for gene delivery in lung cancer therapy

**DOI:** 10.1038/srep21170

**Published:** 2016-02-16

**Authors:** Yu-Fen Wu, Hsi-Chin Wu, Chen-Hsiang Kuan, Chun-Jui Lin, Li-Wen Wang, Chien-Wen Chang, Tzu-Wei Wang

**Affiliations:** 1 Institute of Biomedical Engineering, National Tsing Hua University, Hsinchu, Taiwan; 2 Department of Material Engineering, Tatung University, Taipei, Taiwan; 3 Department of Plastic Surgery, National Taiwan University Hospital, Taipei, Taiwan; 4 Department of Materials Science and Engineering, National Tsing Hua University, Hsinchu, Taiwan; 5 Department of Biomedical Engineering and Environmental Sciences, National Tsing Hua University, Hsinchu, Taiwan

## Abstract

Theranostics, an integrated therapeutic and diagnostic system, can simultaneously monitor the real-time response of therapy. Different imaging modalities can combine with a variety of therapeutic moieties in theranostic nanoagents. In this study, a multi-functionalized, integrated theranostic nanoagent based on folate-conjugated reducible polyethylenimine passivated carbon dots (fc-rPEI-Cdots) is developed and characterized. These nanoagents emit visible blue photoluminescence under 360 nm excitation and can encapsulate multiple siRNAs (EGFR and cyclin B1) followed by releasing them in intracellular reductive environment. *In vitro* cell culture study demonstrates that fc-rPEI-Cdots is a highly biocompatible material and a good siRNA gene delivery carrier for targeted lung cancer treatment. Moreover, fc-rPEI-Cdots/pooled siRNAs can be selectively accumulated in lung cancer cells through receptor mediated endocytosis, resulting in better gene silencing and anti-cancer effect. Combining bioimaging of carbon dots, stimulus responsive property, gene silencing strategy, and active targeting motif, this multi-functionalized, integrated theranostic nanoagent may provide a useful tool and platform to benefit clinicians adjusting therapeutic strategy and administered drug dosage in real time response by monitoring the effect and tracking the development of carcinomatous tissues in diagnostic and therapeutic aspects.

Non-small cell lung cancer (NSCLC) comprises nearly 85% of all lung cancers, and approximately 70% of all newly diagnosed patients present with local advanced or metastatic disease and require systemic chemotherapy^1^. However, it is found that patients often develop drug resistance after long exposure to chemotherapy drugs. Adaptability and sophisticated heterogeneity endow cancer with possibility of developing drug resistance during treatment; thus, reducing therapeutic effect^2^. Gene silencing therapy is superior in cancer treatment for the adjustable sequences of small interference RNA (siRNA), which can specifically reduce different oncogene expression, resulting in reducing the growth of heterogeneous tumor cells^3^. Moreover, siRNA does not lead to genome modification, an important parameter for regulatory and safety considerations. To date, numerous proteins have been targeted by siRNA embedded into non-viral delivery systems in cancer pathologies based on cell cycle, apoptosis, proliferation and angiogenesis pathway studies^4^,^5^,^6^.

Carbon nanodots (C-dots) are nano-scaled carbon materials that inherit with special optical properties due to quantum confinement^7^. As size of carbon nanodots decreases, their band-gap energy increases, resulting in blue-shift photoluminescence. Properties such as high biocompatibility, aqueous dispersity, chemical inertness, and easily functionalized surface make carbon nanodots suitable bioimaging candidates for biomedical applications^8^. Carboxylic groups cover the majority surface of carbon dots. Therefore, molecules that bear amino groups can passivate onto carbon nanodots through amide linkage. Most notable is their potential as replacement for toxic metal-based quantum dots (QDs) currently in use.

Theranostic nanoagent is defined as a carrier with combination of therapeutic and diagnostic application. These nanoagents provide a platform for clinicians to monitor the treatment effect of diseased area by combining diagnostic modality and therapeutic approaches in one system^9^. Diagnostic compartments can provide instant biodistribution of nanoparticles, opportunities in investigating therapeutic mechanism, and adjustment in treatment strategy. Compared with conventional fluorescent dyes and quantum dots, carbon nanodots have great potential in bioimaging because of their consistent photoluminescence without photobleaching, high biocompatibility in living organism, and eco-friendly synthetic process^10^,^11^. By instant monitoring the development of carcinomatous tissue, clinicians can utilize bioimaging molecules to light up tumors and adjust their therapeutic strategy and drug dosage in time for minimizing negative effects.

Gene silencing therapy is one type of personalized medicine, which can down-regulate expression of characterized oncogenic gene and drug resistance related genes through specific interaction of small interference RNA (siRNA) and messenger RNA (mRNA)^12^. However, delivery of siRNA remains a challenging issue in gene silencing therapy due to the fact that siRNA are highly susceptible to nuclease degradation^13^. Passivation enhances photoluminescence of carbon dots and brings additional functions such as the ability to cope with therapeutic agents such as providing positive charges to stabilize and complex nucleic acids^14^,^15^.

Adverse effects during cancer treatment can be reduced by enhancing retention duration and intracellular uptake of nanoparticles in diseased area^16^. It is suggested that enhanced permeability and retention (EPR) effect and endotracheal administration can significantly accumulate nanoparticles at cancerous tissues^17^, while receptor mediated endocytosis through interaction between targeting ligands and receptors on cancer cell surface increases cancer specific cellular uptake of nanoparticles^18^. The specificity of therapeutic molecules to tumor is strongly related to treatment efficiency. Conjugation of targeting motifs on theranostic nanoparticles can increase their local accumulation in cancer tissues. Folic acid (fc), also known as folate, has been widely used in targeting to uncontrolled cancer cell growth. Most of the carcinomas have enhanced folate receptor expression level^19^,^20^.

Lung diseases in general are attractive targets for siRNA-based therapeutics because of their lethality and prevalence^21^. In addition, the lung is anatomically accessible to therapeutic agents via the intrapulmonary route. Accessibility is a key requirement for successful RNAi-based *in vivo* studies, and this characteristic offers several important benefits over systemic delivery, including the use of lower dosage of siRNAs, the reduction of undesirable systemic side effects, and improved siRNA stability due to the lower nuclease activity in the airways compared to the serum. The direct administration of siRNAs into the target organs is a promising strategy for overcoming the problems of intravenous administration. In this study, we have developed a novel theranostic nanoagent that can be used simultaneously in lung cancer diagnosis and therapeutics. In diagnostic segment, photoluminescent carbon dots are synthesized through microwave pyrolysis of glycerol and polyethylenimine (PEI). Free PEI molecules are then conjugated through environmental sensitive disulfide linkage onto PEI-Cdots, forming reducible PEI-Cdots (rPEI-Cdots). The positive charge on rPEI can electrostatically complex with negative charge siRNA molecules. The disulfide linkages between PEI molecules will be broken down by glutathione after theranostic nanoagents being uptaken by cancer cells, and then release therapeutic siRNAs into cytosol to accomplish therapeutic purpose. Folate, serving as tumor targeting molecule, is conjugated on the outmost of rPEI-Cdots/siRNA complex to form fc-rPEI-Cdots/siRNA multifunctional nanoagents. The synthesis route is briefly demonstrated in Fig. 1.

## Results

### Characterization of synthesized fc-rPEI-Cdots compounds

The fc-rPEI-Cdots theranostic nanoagent was synthesized in serial reactions. In brief, PEI-Cdots were synthesized by microwave pyrolysis of glycerol and polyethylenimine (PEI). Then, an equivalent amount of free PEI were conjugated onto PEI-Cdots through Michael addition with N,N’-bis(acryloyl) cystamine (BAC) as a linker, forming reducible PEI-Cdots (rPEI-Cdots). Finally, folic acids were linked to rPEI-Cdots by EDC/NHS coupling, creating multifunctional fc-rPEI-Cdots nanoagent.

**Figure 2:**
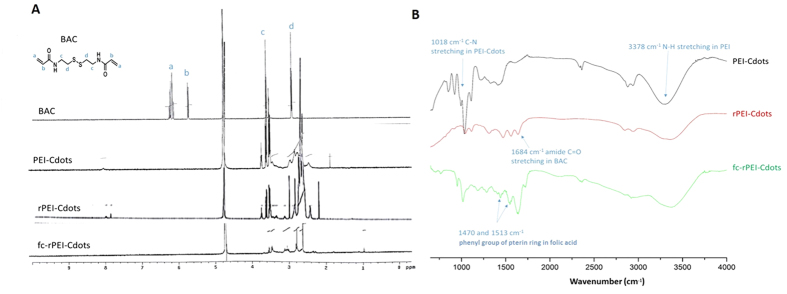
Characterization of fc-rPEI-Cdots by (**A)** H^1^NMR and (**B**) FT-IR. From H^1^NMR, disappearance of alkenyl proton peaks at 5.6 and 6.2 ppm (peak a and b) and preservation of peak at 2.8 ppm (peak d) proved successful synthesis of rPEI-Cdots. Appearance of proton resonance peak between 1.8 ppm showed conjugations of folate. From FT-IR, preservation of C-N and N-H stretching signals of PEI-Cdots (ν = 1018 cm^−1 ^and 3378 cm^−1^), amide C = O stretching (ν = 1684 cm^−1^) signal of BAC, and appearance of phenyl absorption peak of folic acid at 1470 cm^−1^ and 1513 cm^−1^ in the spectrum of fc-rPEI-Cdots demonstrated successful synthesis of fc-rPEI-Cdots.

From ^1^H NMR analysis as shown in Fig. 2A, chemical shifts at 6.2 and 5.6 ppm were contributed from alkenyl protons of N’,N’-bis(acryloyl) cystamine (BAC). The alkyl protons of BAC showed peaks at 2.8 and 3.6 ppm. Peaks between 2–3 ppm and 3–4 ppm were from proton resonances of amine groups in polyethylenimine molecules and protons in carbon nanodots, respectively. Disappearance of alkenyl proton peaks at 6.2 and 5.6 ppm (Fig. 2A,a,b) and preservation of peak at 2.8 ppm from alkyl proton resonance in BAC molecule (Fig. 2A,d) proved successful synthesis of rPEI-Cdots. Appearance of resonance peak at chemical shift 1.8 ppm from methylene proton in folate proved successful conjugation of targeting ligand. Conjugation of folic acid in fc-rPEI-Cdots was also confirmed by FT-IR (Fig. 2B). The characteristic peaks of PEI at 2862–2952 cm^−1 ^and 1453 cm^−1^ were produced by CH_2_CH_2_, at 1122 cm^−1^ by CN bond, and at 1585–1620 cm^−1^ by NH group. Absorption peaks at 3378 cm^−1^ and 1018 cm^−1^, attributed to amine N-H stretching and C-N stretching in PEI-Cdots, were preserved in spectrum of fc-rPEI-Cdots. Absorption peak of amide C = O stretching appeared at 1684 cm^−1^ after synthesis of rPEI-Cdots. Appearance of absorbance peaks at 1470 cm^−1^ and 1513 cm^−1^ in fc-rPEI-Cdots were attributed to phenyl group of pterin ring in folic acid^22^. From ^1^H NMR and FT-IR results, multifunctional fc-rPEI-Cdots nanoagents are suggested to be successfully synthesized by conjugating folate onto reducible PEI-Cdots.

**Figure 3:**
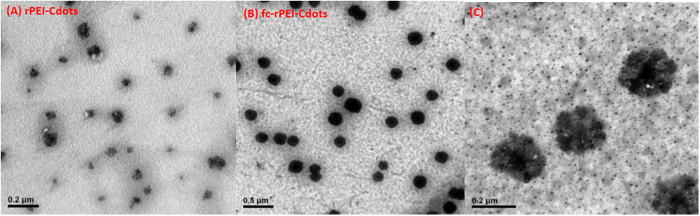
TEM images of (**A**) rPEI-Cdots and (**B**) fc-rPEI-Cdots. rPEI-Cdots and fc-rPEI-Cdots were well dispersed in aqueous solution. The particle size of rPEI-Cdots and fc-rPEI-Cdots was below 200nm. (C) The higher magnification image of fc-rPEI-Cdots in selected area.

The particle size and surface charge of synthesized fc-rPEI-Cdots compounds were measured by dynamic light scattering and zeta potential, respectively (Table 1). Conjugation of free PEI onto rPEI-Cdots not only enlarged particle size from 9.0 ± 1.1 nm to 73.8 ± 0.8 nm, but also increased the surface charge from 4.4 ± 1.7 mV to 11.9 ± 1.9 mV. Particle size and surface charge increased to 143.1 ± 9.9 nm and 25.7 ± 2.3 mV, respectively, after folic acid conjugation. The diameter and charge of fc-rPEI-Cdots became double when compared to rPEI-Cdots. This phenomenon may be resulted from the link-up of two rPEI-Cdots molecules through carboxyl groups in folic acid molecule. After successfully complexed with siRNA, the particle size was slightly increased to 174.3 ± 16.0 nm, while the surface potential was decreased to negative charge around −9  ±  2.1 mV. The complexation with siRNA by fc-rPEI-Cdots had little influence to the particle size, but it strongly reduced the surface charge. The largely reduced surface charge was derived from electrostatic interaction between negatively-charged siRNA and the positively-charged PEI on the surface of fc-rPEI-Cdots. The morphology of fabricated particles was visualized by transmitted electron microscopy (TEM) (Fig. 3). The images showed that the particles were demonstrated in spherical shape with well dispersity in aqueous condition. The images further showed the connection of rPEI-Cdots through folic acid in fc-rPEI-Cdots.

### Photoluminescence property and diagnostic potential of fc-rPEI-Cdots

**Figure 4:**
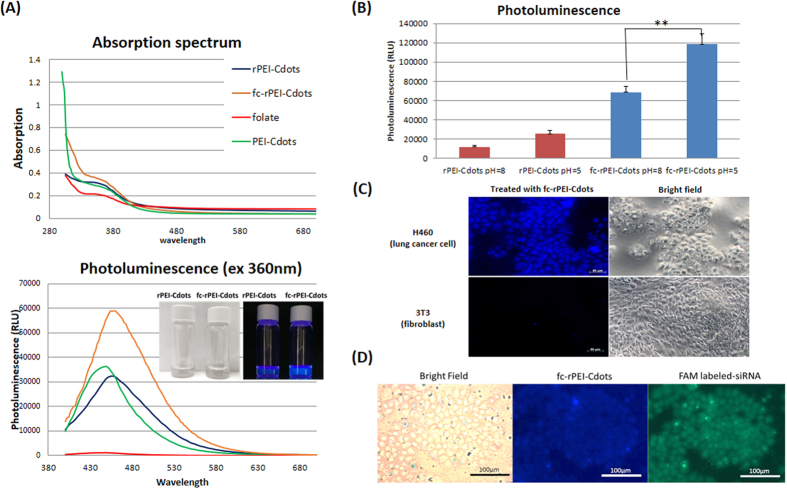
Diagnostic property of fc-rPEI-Cdots nanoparticles. (**A**) Absorption and photoluminescence spectrum of rPEI-Cdots, PEI-Cdots, folate, and fc-rPEI-Cdots. Insert: Photoluminescence (PL) of rPEI-Cdots and fc- rPEI-Cdots under UV excitation. Strongest photoluminescence of fc-rPEI-Cdots appeared at 460 nm when excited by 360 nm. **(B)** The PL intensity of rPEI-Cdots and fc-rPEI-Cdots in different pH environment (pH 5, 8). **(C)** Enhanced accumulation of fc-rPEI-Cdots in H460 could be observed by fluorescent microscopy. **(D)** fc-rPEI-Cdots (blue) and FAM labeled-siRNA (green) were delivered intracellularly.

The unique optical property of fc-rPEI-Cdots provides it the ability of functioning as a bioimaging diagnostic agent. Absorption and emission spectrum of synthesized product were demonstrated in Fig. 4A. The strongest photoluminescence (PL) intensity of each synthesized Cdots-based nanoparticle was observed at 460nm with 360nm excitation. Both absorption and emission spectrums indicate that optical property of carbon nanodots remained the same after serial synthetic processes. Photoluminescence of fc-rPEI-Cdots and rPEI-Cdots with visible blue light emission under UV excitation as clearly demonstrated in the inserted figure (Fig. 4(A)). We are also interested in knowing that if pH value has any influence in the photoluminescence intensity of different polymer passivated Cdots nanoparticles. The results showed that the intensity of rPEI-Cdots and fc-rPEI-Cdots were all increased significantly with the pH change from 8 to 5, especially in folate conjugated group (Fig. 4B). This finding would be advantageous when these nanoparticles were uptaken by cancer cells followed by traveling into late endosome or lysosome, where the pH value was close to pH 5.5.

Cellular uptake of fc-rPEI-Cdots could be observed by fluorescence microscopy. Enhanced cellular uptake of fc-rPEI-Cdots in lung cancer cells was observed when compared to normal fibroblasts (Fig. 4C). This selectively enhanced accumulation in cancerous cell was derived from receptor mediated endocytosis. Over-expression of folate receptors on the surface of H460 induced endocytosis of fc-rPEI-Cdots through the interaction of folate and its receptors on cell membrane. Also, cellular uptake of fc-rPEI-Cdots/FAM-siRNA complex was observed in Fig. 4D. With the labeling of fluorescein amidite (FAM), the intracellular release of siRNA could be detected by examining green fluorescence and in agreement with the co-localization of blue emission light of our nanoparticles. It is also noted that both siRNA and fc-rPEI-Cdots were well dispersed intracellularly.

### Therapeutic siRNA loading and redox sensitivity of fc-rPEI-Cdots

**Figure 5:**
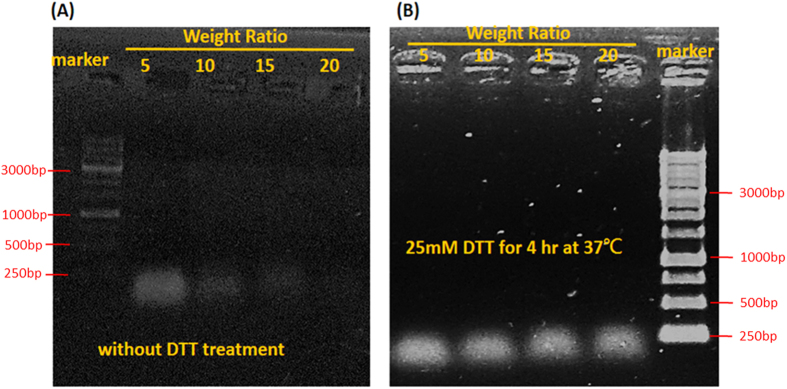
Electrophoresis of fc-rPEI-Cdots/siRNA nanocomplex with different weight ratio of 5, 10, 15, and 20 under normal (without DTT treatment (**A**)) and reductive environment (with 25mM DTT reducing agent treated for 4 hours at 37 °C (**B**)). The encapsulated siRNA could complex with fc-rPEI-Cdots compactly at weight ratio above 15 and be released in reductive environment. (Weight ratio is defined as the weight of nanoparticles versus the weight of siRNA. The weight of siRNA can be derived and calculated from the measurement of OD value).

The ability of fc-rPEI-Cdots encapsulating nucleic acids was studied by gel electrophoresis. Different weight ratios of fc-rPEI-Cdots and small interference RNA (siRNA) were mixed together to form fc-rPEI-Cdots/siRNA complex. To mimic the redox microenvironment in cytosol, a reducing agent, dithiothreitol (DTT), was used to cleave disulfide linkages in fc-rPEI-Cdots. As shown in Fig. 5A, siRNA could successfully complex with fc-rPEI-Cdots at weight ratio above 15. After reducing of disulfide linkages by DTT treatment, encapsulated siRNA cargo were released from fc-rPEI-Cdots/siRNA complexes. These results suggest that fc-rPEI-Cdots could form compact complex with siRNA and release siRNA in reducing environment above weight ratio of 15. In the following *in vitro* study, fc-rPEI-Cdots/siRNA complex with such weight ratio were used.

### *In vitro* study

#### Biocompatibility of fc-rPEI-Cdots and therapeutic effect of fc-rPEI-Cdots/pooled siRNA

**Figure 6:**
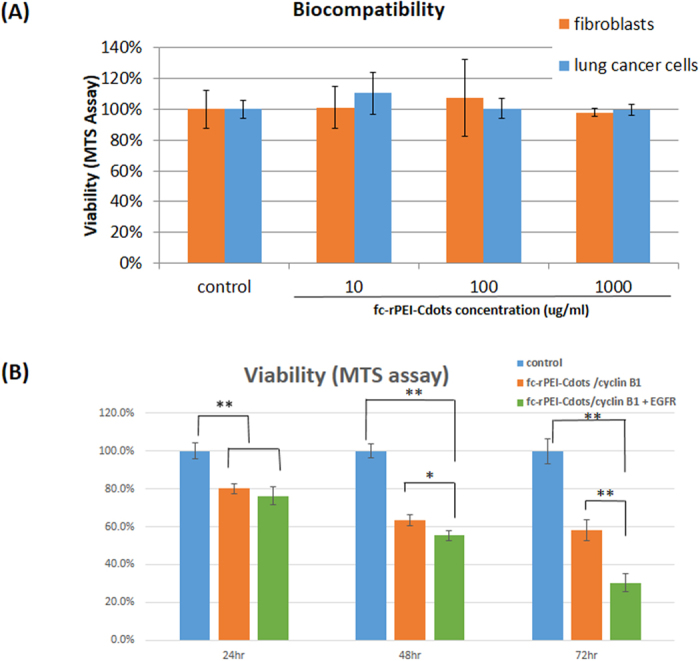
Biocompatibility and therapeutic effect of fc-rPEI-Cdots/siRNA complexes. (**A**) Biocompatibility of fc-rPEI-Cdots in human lung cancer cells and mouse embryo fibroblasts after incubated with fc-rPEI-Cdots for 24 hours. Viability of each group remained above 90% suggests that fc-rPEI-Cdots is a non-cytotoxic and biocompatible material. (**B**) Viability of lung cancer cells (H460) after treated with fc-rPEI-Cdots/single siRNA (cyclin B1) and rPEI-Cdots/pooled siRNA (cyclin B1 + EGFR) for 24 hours, 48 hours, and 72 hours. Viability of H460 dropped to 30% after treated with fc-rPEI-Cdots/pooled siRNA after 72 hours. (*p < 0.05 ; **p < 0.01, N = 4).

The non-small cell lung cancer cells (H460) and mouse embryo fibroblasts (3T3) were treated with different concentrations of fc-rPEI-Cdots to examine the cytotoxicity of our synthesized multifunctional nanoagent. As shown in Fig. 6A, cell viability of test groups treated from low to high concentrations of fc-rPEI-Cdots all remained above 95% compared to non-treated control. This high viability suggests that our fc-rPEI-Cdots multifunctional nanoagent is a highly biocompatible material unlike conventional quantum dots that may mediate cytotoxicity to living cells.

Therapeutic effect of our synthetic fc-rPEI-Cdots/pooled siRNA (cyclin B1 plus EGFR) and fc-rPEI-Cdots/single siRNA (cyclin B1) nanoagents was examined by measuring viability of lung cancer cells after treatment. Viability of H460 lung cancer cells was reduced to 75~80% after treated with fc-rPEI-Cdots/pooled siRNA or fc-rPEI-Cdots/single siRNA for 24 hours, respectively. The therapeutic effect of fc-rPEI-Cdots/pooled siRNA complexes lasted for 72 hours, reducing the survival of lung cancer cells to 30% while fc-rPEI-Cdots/single siRNA complexes maintained therapeutic effect at 60% (Fig. 6B). The combinational synergistic effect was therefore revealed and supported by MTT data.

**Figure 7:**
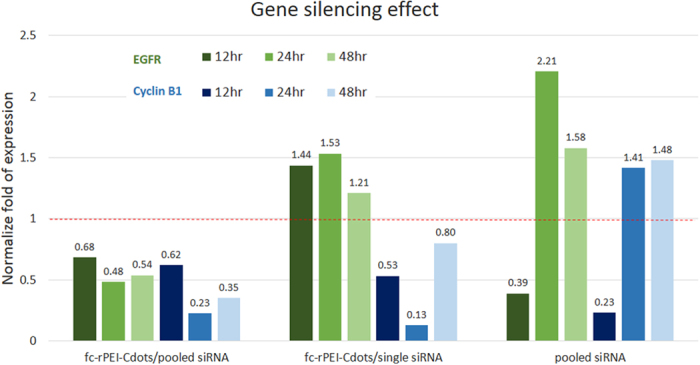
Gene silencing effect of EGFR and Cyclin B1 after treated with fc-rPEI-Cdots/pooled siRNA, fc-rPEI-Cdots/single siRNA, and pooled siRNA (without nanocarrier) in H460 for 12 hours, 24 hours, and 48 hours. The group of pooled siRNA showed transient gene silencing effect in 12 hours, and failed to reduce gene expression thereafter. On the other hand, fc-rPEI-Cdots/pooled siRNA showed sustained gene silencing effect for prolong period of time in 48 hrs. (N = 4).

The gene silencing effect of cyclin B1 and epidermal growth factor receptor (EGFR) genes was quantified by qPCR in Fig. 7. The mRNA gene expressions of cyclin B1 and EGFR were all suppressed lower than one fold in fc-rPEI-Cdots/pooled siRNA group from 12 to 48 hrs. On the other hand, if naked siRNA including cyclin B1 and EGFR was transported without nanoparticles serving as delivery vehicle, the inhibitory effect was transient for 12 hrs and then surged back to normal level more than one fold when compared to the without treatment control group. This suggests that fc-rPEI-Cdots may promote the generic materials uptake by lung cancer cells and prevent siRNA degradation in culture medium. It is interesting to note that the gene expression of cyclin B1 and EGFR in fc-rPEI-Cdots/single siRNA was obviously different. Since only cyclin B1 siRNA was used in this delivery system, the inhibitory effect of this gene expression was noticed, while the secretion level of EGFR was not influenced. We also found that only fc-rPEI-Cdots/pooled siRNA complex could sustain gene silencing ability for 48 hours when compared to other two conditions. This phenomenon demonstrated the inhibitory effect of fc-rPEI-Cdots/pooled siRNA for sustained therapeutic effect may be achieved.

### *In vivo* study

**Figure 8:**
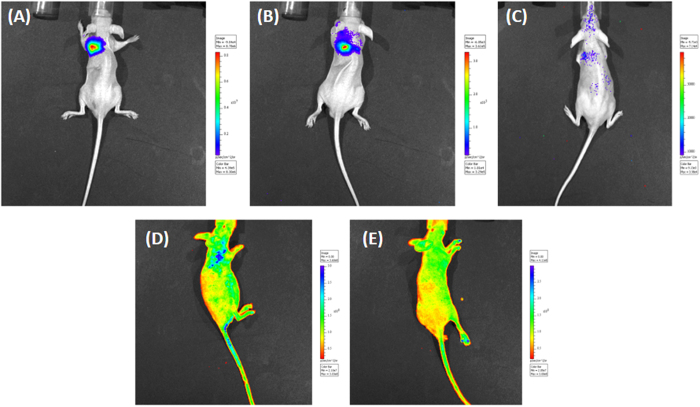
Monitoring luciferase inhibition *in vivo* with bioluminescent imaging. Representative images show the reduction in lung tumor size following intratracheal instillation of fc-rPEI-Cdots nanoagents in luciferase-expressing H460 lung carcinoma. Panels (**A**–**C)** depict bioluminescent images of the lungs before and after treatment. (**B**) 7 days, (**C**) 10 days after two times inhaled administration. After aerosol delivery, the fc-rPEI-Cdots/pooled siRNA nanoagents (**D**) can accumulated at lung region when compared to PBS (**E**).

In a further study, the activity of fc-rPEI-Cdots/pooled siRNA complexes against the growth of H460 tumor in nude mice was investigated. When the tumor reached to approximately 200 mm^3^, fc-rPEI-Cdots/pooled siRNA or PBS was respectively delivered by inhalation into the lungs every other day for two times. The efficient and homogeneous distribution of the fc-rPEI-Cdots nanoagents to most parts of the lung tissue is a prerequisite for studying target-specific siRNA efficacy. The endotracheal application of suspended fc-rPEI-Cdots nanoagents by a MicroSprayer^TM^ can result in nanoparticle deposition in smaller airway and peripleural tumor cells. To estimate whether the aerosol delivery of our fc-rPEI-Cdots nanoagents had a valid gene-silencing effect on the lung tumor, the mice were treated with a luciferase expression H460 cells. The average size of H460 tumor became statistically smaller at day 7 and 10 (P < 0.001) for the fc-rPEI-Cdots/pooled siRNA -treated group compared to the group treated by PBS. In mice receiving fc-rPEI-Cdots/pooled siRNA nanoagents, bioluminescence intensity was inhibited by 50% when compared with bioluminescence before treatment (Fig. 7A–C). In addition, the siRNA effect of the platform would continue for at least 14 days. We also found that after aerosol delivery, the fc-rPEI-Cdots/pooled siRNA nanoagents can accumulated at lung region when compared to PBS, demonstrating the bioimaging property of our nanoparticles (excitation 360 nm/ emission 460 nm) (Fig. 8D,E).

## Discussion

PEI and glycerol were mixed together and pyrolyzed by microwave for PEI-Cdots synthesis. In our experience, pyrolysis duration affected the concentration of PEI-Cdots in the solution, but not the sizes of PEI-Cdots. Concentration of PEI-Cdots reached maximum after ten minutes pyrolysis, emitting strongest intensity of photoluminescence when compared to the products synthesized from different pyrolysis duration. Bio-reducible disulfide bonds were then introduced into our multifunctional fc-rPEI-Cdots nanoagents through one-step Michael addition^23^. Nucleophilic amine groups on the surface of PEI-Cdots and PEI molecules interacted with alkenyl groups of BAC molecule, a disulfide bond-containing molecule, forming reducible PEI-Cdots (rPEI-Cdots). The results showed that one-step synthesis was efficient and reduced the loss of product during purification process. PEI conjugation increased surface charge of rPEI-Cdots, which also enhance proton sponge effect and the endosomal escape ability. In the molecular structure of BAC as shown in Fig. 2A, there is disulfide bond (S-S) in it. After linking free PEI molecules by BAC, the disulfide bond can be reduced into thiol groups (-SH). This will result in the cleavage and separation of large PEI molecule into smaller one. With the decrease in positive charge, the binding affinity with siRNA decrease ,and then may facilitate the release of siRNA. In our previous study, it has been suggested that inserted disulfide bonds could be cleaved by glutathione in cytosol and release encapsulated therapeutic biomolecules (*viz.*, anti-cancer drugs, genetic materials)^24^. Also, cleavable rPEI-Cdots results in small molecule weight PEI with lower cytotoxicity because of weak electrostatic interaction between cellular organelles membrane and PEI molecules.

Folic acids were conjugated onto rPEI-Cdots through EDC/NHS coupling reaction. However, both amine group and carboxyl group existed in folic acids. Conjugation of folic acid through EDC/NHS coupling may result in folic acid chain. Therefore, protection of amine groups in folic acid by tert-butyl bicarbonate (t-BOC) is required and demonstrated for avoiding folic acid chain in our study^25^,^26^. After that, t-BOC was removed by t-BOC removal agent from theranostic fc-rPEI-Cdots. Consequently, the multifunctional nanoagent was successfully synthesized and characterized by ^1^H NMR and FT-IR (Fig. 2).

The research work demonstrated by Wang Q, *et al.* also suggest Cdots-based and PEI-adsorbed complexes can be utilized as gene delivery carrier^27^. In our work, reducible PEI was introduced for the purpose of cleavage of PEI into small molecules for the enhancement of siRNA release; thus, promoting gene silencing effect as shown in Fig. 7. The active targeting ligand, folic acid, was also conjugated for increasing the specificity and localizing to cancer cells. In addition, our theranostic fc-rPEI-Cdots NPs were successfully administered through aerosol delivery for the bioimaging purpose and therapeutic effect in *in vivo* orthotopic lung cancer model.

Synthesized PEI-Cdots had a diameter of 9 nm, similar to the size of polyethylenimine passivated carbon dots in other studies^28^. Passivation of PEI not only enhanced the photoluminescence intensity but also provided functional cationic amine groups on the surface of PEI-Cdots to complex with siRNA (Figs 4 and 5). Since BAC and excessive amount of cationic PEI molecules were incorporated in the system to form rPEI-Cdots, increased particle size and surface charge were measured as expected (Table 1). Spherical morphology and well aqueous dispersity of fc-rPEI-Cdots were observed by transmitted electron microscopy (Fig. 3). A closer observation of fc-rPEI-Cdots by TEM showed that several condensed rPEI-Cdots were connected together after folic acid grafting. Even so, particle size of synthesized nanocarrier was still kept below 200 nm, a critical particle size for endocytosis. Complexing with negatively charged siRNA significantly reduced the surface charge of fc-rPEI-Cdots as shown in Table 1.

Absorption and photoluminescence spectrum were measured to examine the bioimaging potential of synthesized fc-rPEI-Cdots. Each fabricated material possessed an absorption peak at 360 nm and the strongest photoluminescence peak appeared at 460 nm (Fig. 4). Similar intensity shown in optical spectrum suggests that microwave fabrication process has little impact on photoluminescence property of carbon nanodots. Passivation of PEI disrupted sp^2^ hybridization by nitrogen doping on the surface of PEI-Cdots as excitation center of electron-hole pairs for the energy recombination^29^. Conjugation of other molecules onto rPEI-Cdots didn’t influence nitrogen doping on the surface of PEI-Cdots core^30^; therefore, each fabricated product shared similar optical property. However, changes in particle size during fabrication process affect strongly on the intensity of photoluminescence because of the shielding effect on emission light by conjugating PEI and folic acids. Despite the reduced intensity of emission light, blue photoluminescence of fc-rPEI-Cdots was still detectable by naked eye. Also, it is interesting to mention that fc-rPEI-Cdots emit stronger photoluminescence in acidic environment. This observed phenomenon was due to the increased dispersity of nanoparticles in acidic environment and may take advantages when applied in cancer tissue region. Ionic strength and pH values are known to affect the fluorescence properties of different molecules and nanoparticles. A dependence of the C-dot PL intensity on the pH value was seen in some studies^31^,^32^. Polyethylenimine on the surface of fc-rPEI-Cdots absorbed protons in acidic environment, resulting in enhanced surface charge of fc-rPEI-Cdots. Electrical repulsion made fc-rPEI-Cdots disperse uniformly in acidic aqueous solution and reduce the chance of light emission shielded by particle aggregation. This unique property makes fc-rPEI-Cdots as lung cancer imaging molecules for the fact that fc-rPEI-Cdots can emit stronger photoluminescence in acidic endosome after receptor mediated endocytosis by lung cancer cells.

It is noteworthy to mention that fc-rPEI-Cdots was highly accumulated in lung cancer cells when compared to normal fibroblasts as observed in Fig. 4C. Also, fc-rPEI-Cdots/siRNA complex showed detectable blue photoluminescence in *in vitro* cell culture study. After uptake, siRNAs were released from fc-rPEI-Cdots/siRNA complexes due to disulfide bond cleavage by DTT, reductive agent with similar behavior like glutathione in cytosol, as uniformly dispersed with green fluorescence of FAM-labeled siRNA.Gene silencing therapy can inhibit different tumor types and subtype growth from adjustable sequences and specifically reduce oncogene expression. In the context of gene therapy applied to cancers, siRNA unlike antibodies or tyrosine kinase inhibitors that respectively react only with surface antigens and tyrosine kinase protein acts as a loss-of-function strategy that can inhibit virtually every single protein of interest regardless to its localization within the cells. These small entities aim to modulate the expression of overexpressed or mutated genes identified as a key hurdle. In this study, siRNA encapsulating ability of fc-rPEI-Cdots were examined by electrophoresis as shown in Fig. 5, demonstrating our fc-rPEI-Cdots/siRNA multifunctional nanoagents could release siRNAs through cleavage of disulfide bonds in reducing environment. Relatively high concentration of biological reducing glutathione in cytosol provides the potential of cleaving disulfide bonds intracellularly^33^. The prerequisite of delivery system is to favour cellular uptake and target only tumor site and tumoral cells, limit side effects, and improve the therapeutic efficacy. In comparable with passive clustering, active targeting is more and more envisaged to enhance the preferential tumor region accumulation and avoid cytotoxic adverse effect. This can be conducted by grafting on the surface of the delivery vehicle with specific markers or ligands that are only expressed or at least overexpressed in tumors. For this purpose, chemical modification and conjugation of peptide and antibody molecules on polymers have been favoured. In this study, receptor mediated endocytosis induced by folate ligand fixation leads to a facilitated internalization of siRNA delivery into cytoplasmic vesicles through cascade signaling pathway. After internalization, early endosomal vesicles are formed and matured in late endosomes characterized by an acidic pH and finally fuse with lysosome vesicles^34^. In these endosomes, the degradation of nanocarriers can be initiated and nucleic acids are then released through endosomal escape, which is a critical step for an efficient cytoplasmic siRNA delivery.

Biocompatibility of our bare multifunctional fc-rPEI-Cdots nanoagents was confirmed. Both normal fibroblast and lung cancer cells showed high viability, above 90%, after treated with fc-rPEI-Cdots for 24 hours. These high biocompatibility and lung cancer targeting properties provided our multifunctional fc-rPEI-Cdots nanoagent versatility in lung cancer gene delivery applications (Fig. 6A).

Lung cancer therapeutic effect was also observed by measuring viability of H460 cells as demonstrated in Fig. 6B. Both fc-rPEI-Cdots/cyclin B1+ EGFR and fc-rPEI-Cdots/cyclin B1 complexes showed anti-cancer effect in treatment period for 72 hrs. Survival rate of lung cancer cells was reduced to 80% after 24 hrs administration and further to 60% after 48 hrs. After that, the viability was lower down to 30% in fc-rPEI-Cdots/pooled siRNA group at 72 hrs, while fc-rPEI-Cdots/single siRNA group was only maintained at 60%, suggesting the synergistic effect of two therapeutic genes. Compared with gene silencing effect as demonstrated in Fig. 6, fc-rPEI-Cdots/ pooled siRNA could show growth arrest in gene senescence expression for the entire period, while fc-rPEI-Cdots/single siRNA only took effects on cyclin B1 gene. The gene expression of EGFR was not influenced. For the pooled siRNA group (without carrier), the inhibitory effect on both EGFR and cyclin B1 gene expressions was temporary for 12 hrs. This phenomenon may result from different cellular uptake mechanism of fc-rPEI-Cdots/ pooled siRNA and pooled siRNA. Internalization of fc-rPEI-Cdots/pooled siRNA through folate-receptor mediated endocytosis increased the affinity of folate ligand to nearby folate transporter and substantially enhance the efficiency of delivery of fc-rPEI-Cdots/pooled siRNA complexes. Because of sequentially enhanced ligand-receptor affinity of folate to folate receptor, fc-rPEI-Cdots/pooled siRNA complexes showed sustained gene silencing and therapeutic effect^35^,^36^.

The success of the delivery of RNAi-based therapeutics requires efficiency, convenience, and patient compliance with the delivery route. The direct-delivery approach can overcome barriers in clinical testing, and successful siRNA can offer high specificity to specific organs and fewer undesirable off-target effects compared to systemic administration. For these reasons, the aerosol delivery system of siRNA agents is a safe and powerful potential treatment for lung cancer; because the anatomical structure and location of the lungs make this simple, non-invasive approach possible and its high delivery efficiency reduces systemic side effects. RNA interference currently offers new opportunities for gene therapy by the specific extinction of targeted genes in cancer diseases. However, the main challenge for nucleic acid delivery still remains its efficacy through intravenous administration. In our study, inhalation delivery to bypass the obstacles of intravenous injection has been developed and optimized to encapsulate siRNA and to specifically promote their delivery into tumor cells and improve their pharmacokinetics for anti-cancer purposes.

## Conclusions

In this study, we have successfully synthesized a novel multifunctional theranostic fc-rPEI-Cdots/ siRNA nanoagent. In diagnosis modality, carbon dots are promising bioimaging agents for their persistent photoluminescence, high biocompatibility, and eco-friendly production process. In therapeutic segment, gene silencing therapy knockdown oncogenetic signaling pathway and induce apoptosis through specific interaction of siRNA and corresponding mRNA. Moreover, this specificity can promote treatment efficiency by targeting to personalized genetic defects in the tumor. In lung cancer therapy, folate are conjugated as targeting ligand for the fact of folate receptors over-expression on the surface of cancer cells.

Our theranostic nanoagents absorb 360 nm and emit 460 nm, the wavelength of blue light. These nanoagents have size of 143 nm and disperse well in aqueous solution as shown in TEM images. Their positive surface charge can complex with negatively charged siRNAs. The fc-rPEI-Cdots could function as a siRNA carrier and release siRNA in reducing environment as shown in electrophoresis. Also, enhanced accumulation of fc-rPEI-Cdots is observed in lung cancer cells compared to normal fibroblasts. Viability of H460 treated with fc-rPEI-Cdots/ pooled siRNA complex for 3 days is significantly reduced to nearly 30%. The gene expression of cyclin B1 and EGFR expression are also obviously inhibited. In conclusion, our novel theranostic fc-rPEI-Cdots/ siRNA nanoagents have a potential in lung cancer targeting and treatment.

## Materials/Methods

*Materials:* Glycerol (> = 98%), bis-acryloyl cysteamine, sodium phosphate, sodium bicarbonate, N-hydroxysuccinimide (NHS), and dimethyl sulhpoxide (DMSO) were purchased from Sigma. Branched polyethylenimine (MW = 600 Da) and di-tert-butyl dicarbonate (tBOC) were purchased from Alfa. Folic acid was purchased from ACROS. Sodium chloride, potassium phosphate monobasic, and potassium chloride were obtained from J.T. Baker. Each chemical was used without further purification after received. 10 kb DNA ladder was kindly provided by GeneDireX. Dulbecco’s Modified Eagle Medium (high glucose) and penicillin-streptomycin were obtained from Gibco as powder form. RPMI-1640 was purchased from Biosera in solution. Fetal bovine serum was purchased from Biolodical Industries. PureLink RNA Mini Kit, RNA Extraction kit was purchased from Amnion. CellTiter 96^@^Aqueous One solution Cell proliferation assay (MTS assay) was purchased from Promega. iSCRIPT cDNA synthesis kit and iQ SYBR Green Supermix were purchased from Biorad for qPCR analysis. Cellulose dialysis bag (MWCO = 1000 Da) was purchased from Orange. siRNAs were designed and purchased from GenePharama. Sequences of siRNAs target to cyclin B1 and EGFR are 5′-GGCGAAGAUCAACAUGGCATT-3′ and 5′-CACAGUGGAGCGAAUUCCUTT-3′, respectively. Each was modified with 2′-O-methyl group.

### Synthesis of fc-rPEI-Cdots

The 10 ml of glycerol was mixed with 0.5 g PEI in 6 ml 10 mM phosphate buffer, and pyrolyzed by 550W microwave for 5 min to synthesize PEI-Cdots. Next, bisacryloyl cysteamine (BAC) was weighted and dissolved in 5 ml methanol, mixing with PEI-Cdots and free PEI aqueous solution. The reaction of BAC, PEI and PEI-Cdots mixture continued at 50 °C for 24 hr. After that, unreacted reactants were exterminated by dialyzing against diH_2_O in a dialysis bag (MWCO = 1000 Da). The synthesized rPEI-Cdots were collected by lyophilizing the dialyzed solution. Before conjugating folate on rPEI-Cdots, amine groups on folates were capped by di-tert-butyl dicarbonate (tBOC) to prevent from unexpected conjugation process. In brief, 20 mg folates were mixed with appropriate amount of 1-Ethyl-3-(3-dimethylaminopropyl) carbodiimide (EDC) and N-hydroxysuccinimide (NHS) along with 10 mg tBOC in 10 ml DMSO. The reaction proceeded for 24 hours. Unreacted reactants were removed and fc-tBOC molecules were collected by dialysis (MWCO =  3000 Da) against alkaline solution. Finally, 10 mg fc-tBOC, 20 mg rPEI-Cdots and EDC/NHS were dissolved in 10 ml DMSO for crosslinking these two segments of polymer. The reaction continued at room temperature for 1 day. Then, the solution was dialyzed against 1M NaOH overnight to eliminate unreacted folic acids. The final product of fc-rPEI-Cdots powder was collected by lyophilization.

### Physiochemical properties characterization

5 mg of samples were dissolved in 1 ml D_2_O. Then, the mixed solution was sonicated for 10 mins before they were analyzed by Varian Unity INOVA 500 NMR Spectrumeter, FT-IR (PerkinElmer, Vertex 80v) and dynamic light scattering (Nano-ZS ZEN3600 MALVERN Instrument) to detect the successful conjugation of fc-rPEI-Cdots, particle size and zeta potential, respectively. To prepare TEM specimen, nanoparticle suspension was negatively stained with 2% (w/v) phosphotungstic acid. Both nanoparticle suspension and phosphotungstic acid solution were dropped on carbon-coated copper grids and air-dried in room temperature. TEM image was observed and taken using Hitachi H7500 (Hitachi, Japan) at different magnifications. The absorption and photoluminescence spectrum of synthesized materials were obtained by UV-Vis spectrophotometer (Varian, Palo Alto, CA, USA) measurement. Absorption values were measured every 5 nm between wavelengths of 280 nm to 800 nm. Photoluminescence spectrum was obtained under excitation of 360 nm UV light, the wavelength of absorption peak. Photoluminescence of fc-rPEI-Cdots and rPEI-Cdots in different pH environment was measured by ELISA Reader (PerkinELmer Victor X). 5 mg of fc-rPEI-Cdots and rPEI-Cdots was dissolved separately in 1 ml di-water and sonicated for 30 minutes. Then pH value of each solution was adjusted to 8 and 5 by 1N HCl and 1N NaOH. Afterwards, 200 ul of each solution in different pH value was extracted and placed in a black plate for measuring photoluminescence by ELISA Reader. (The excitation wavelength was 360 nm and the emission wavelength was 460 nm).

### Nucleic acid encapsulating ability and redox property

siRNA were mixed with fc-rPEI-Cdots by weight ratios of 5, 10, 15, and 20 (weight ratio is defined as the weight of nanoparticles versus the weight of siRNA. The weight of siRNA can be derived and calculated from the measurement of OD value). Afterwards, the mixed solutions were placed under room temperature for 30 minutes for complex formation. Then, dithiothreitol (DTT) was added into complex solution for 4 hours to examine the redox property of disulfide linkage (The final concentration of DTT was 25 mM). Electrophoresis was then used to observe the nucleic acid encapsulation and release abilities of fc-rPEI-Cdots.

### Biocompatibility and cellular uptake of fc-rPEI-Cdots

8 × 10^3 ^cells of 3T3 (mouse embryo fibroblasts) and H460 (human non-small cell lung cancer cells) were pre-seeded in 96-well plate overnight. Before treatment, medium was replaced with low serum medium (DMEM + 2% FBS + 1% PS) for cell starvation. After 24 hours, the medium with different concentrations of fc-rPEI-Cdots was added to investigate therapeutic effect of our synthetic nanoparticles. Cells were incubated with fc-rPEI-Cdots for 24hr. Viability of cells was examined by MTS assay measuring absorption at 490 nm after 1.5 hours incubation with cells for biocompatibility test. Equation of calculating viability is listed below.


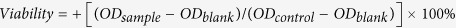


For observation of cellular uptake of fc-rPEI-Cdots nanoparticles, 8 × 10^3 ^cells of 3T3 (mouse embryo fibroblasts) and H460 (human large cell lung cancer cells) were pre-seeded in 96-well plate overnight. Cell starvation was also carried out prior to treatment. Fc-rPEI-Cdots at concentration of 1000 mg/ml was then added, co-incubated with 3T3 and H460 cells, respectively. Selective uptake of fc-rPEI-Cdots in lung cancer cells was observed by fluorescence microscopy.

### Intracellular uptake of fc-rPEI-Cdots/siRNA

2.5 × 10^4^ H460 cells were seeded in 4-chamber slide overnight. After starvation for 24 hrs, medium was replaced with fc-rPEI-Cdots/FAM-siRNA complex (weight ratio = 15) containing growth medium. Treatment of fc-rPEI-Cdots/FAM-siRNA was lasted for one day. After that, medium was replaced with 1 ml of 4% formaldehyde to fix cells. After washing three times by phosphate buffer saline, mounting medium was then added to replace formaldehyde and sealed the slide. The slide was observed by fluorescence microscopy under UV light and blue light excitation for locating fc-rPEI-Cdots and FAM-labeled siRNA, respectively.

### *In vitro* gene silencing effect of fc-rPEI-Cdots/siRNA

2.5 × 10^4 ^H460 lung cancer cells were pre-seeded in 24-well plate overnight, and starved in low serum medium (DMEM + 2% FBS + 1% PS) the following day. After one day starvation, fc-rPEI-Cdots mixed with pooled siRNA (cyclin B1 siRNA mixed with EGFR siRNA at 1:1 ratio) at weight ratio of 15 was added in low serum medium. Cells were incubated with fc-rPEI-Cdots/siRNA for 24 hr. Trizol was added into wells to lyse treated cells for total RNA extraction. Then, lysed solution was mixed with chloroform and centrifuged at 4 °C to collect RNA pellet. 75% ethanol was then added to dissolve RNA pellet for removing proteins. Washed RNA pellet was collected by centrifuge and dried. Then, RNA was dissolved in RNase-free water. RNA solution was then mixed with 5 ul iScript Reverse Transcription Supermix. The mixed solution was incubated in the following process, 5 min at 25 °C, 30 min at 42 °C, followed by 5 min at 85 °C. The cDNA solution was preserved at −80 °C fridge. Gene expressions were examined by the following procedures. cDNA samples were collected and mixed with GoTaq qPCR Master Mix and primers to examine expression level of different genes. Primer sequences of cyclin B1 (Forwards: 5′-AGTAAAAGTCTACCACCGAATCC-3′, Reverse: 5′-ACTTAGAATTATGGCAGCAATCAC-3′), EGFR (Forwards: 5′-AGTAAAAGTCTACCACCGAATCC-3′, Reverse: 5′-GGTGGATATTGACTAGGAGAG-3′), and GAPDH (Forwards: 5′-AAGGTCGGAGTCAACGGATT-3′, Reverse: 5′-GGCAACAATATCCACTTTACCAGA-3′) were optimized prior to experiments. Data was collected by Miniopticon Real Time PCR machine. Normalized folds of gene expression were calculated by the following equations.


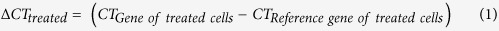



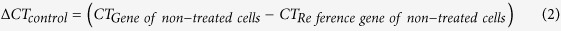



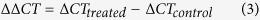


Normalized fold of gene expression = 2^−(ΔΔCT)^.

### Establishment of orthotopic lung cancer model and anticancer effect of theranostic fc-rPEI-Cdots/siRNA

Male athymic nude mice were purchased from the National Laboratory Animal Center, Taiwan. All animal experiments were performed in accordance with and approved by the Institutional Animal Care and Use Committee at the National Tsing Hua University. The mice were used in accordance with institutional guidelines when they were 6–10 weeks old. The human non-small cell lung carcinoma cell line with luciferase transfection (H460-luc) was supported and a kind gift from Prof. Leaf Hung, University of North Carolina at Chapel Hill, USA. Briefly, H460 human lung adenocarcinoma epithelial cells transfected with luciferase (2  × 10^6^) were resuspended in 50 μl of RPMI medium containing 20% FBS and administered intratracheally to the murine lung through a catheter and MicroSprayer^TM^ aerosolizer (Penn-Century, Inc., USA). The progression of tumor growth was monitored by an IVIS (Caliper Life Sciences) imaging system at predetermined time intervals. In details, animals were anesthetized with isoflurane using the XGI-8 Gas Anesthesia System. 150 mg/kg of D-luciferin substrate was injected intraperitoneally to tumor-implanted mice prior to be monitored. The region of interest (ROI) in tumor area were selected and further used for quantification by the software provided by the Caliper Life Sciences. The fc-rPEI-Cdots/siRNA nanoagents were administered in H460-luc orthotopic lung tumor models at 3 weeks after implantation, at which time the primary tumor has become established. In the drug administration study, the administration volume was 50 μl and final concentration of fc-rPEI-Cdots and siRNA were 75 μg/ml and 5 μg/ml, respectively. The therapeutic nanoagents were inhaled by using a MicroSprayer^TM^ aerosoliser. Data are from a representative experiment of four independent experiments.

### Statistical Analysis

All data are expressed as mean ± standard error of the mean unless otherwise indicated. The significance of the effect of selected parameters on the outcome variables was analyzed by multifactor analysis of variance (ANOVA). Group comparisons were made by Fisher’s PLSD. Statistical significance was accepted at a level of p < 0.05.

## Additional Information

**How to cite this article**: Wu, Y.-F. *et al.* Multi-functionalized carbon dots as theranostic nanoagent for gene delivery in lung cancer therapy. *Sci. Rep.* **6**, 21170; doi: 10.1038/srep21170 (2016).

## Figures and Tables

**Figure 1:**
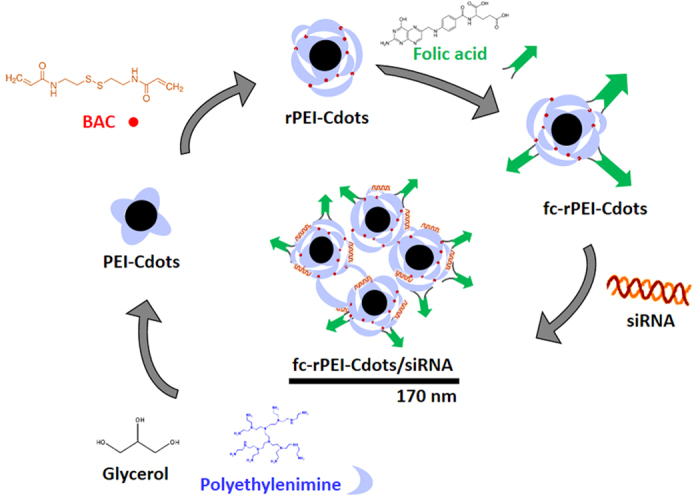
Synthesis route of fc-rPEI-Cdots/siRNA multifunctional nanoagents. In this study, fc-rPEI-Cdots nanoagent were synthesized through a serial process. Microwave pyrolysis was first carried out to fabricate PEI-Cdots. Then, rPEI-Cdots were formed by conjugation of excess PEI molecules to PEI-Cdots through Michael addition. Afterwards, folic acids were attached to rPEI-Cdots to develop fc-rPEI-Cdots nanoagnet. With the possitive charge on the surface, our fc-rPEI-Cdots naonagent can compex with negatively charged therapeutic siRNA molecules.

**Table 1:** Particle size and surface charge measurement of different polymeric constituents.

Sample	Particle size^A^ (d,nm)	Surface charge^B^ (mV)
PEI-Cdots	9.0 ± 1.1	4.4 ± 1.7
rPEI-Cdots	73.8 ± 0.8	11.9 ± 1.9
fc-rPEI-Cdots	143.1 ± 9.9	25.7 ± 2.3
fc-rPEI-Cdots/siRNA (weight ratio = 15)	174.3 ± 16.0	−9 ± 2.1

(**A**) Particle size were measured by dynamic light scattering (DLS).

(**B**) Surface charge was determined by zeta potential.
